# Pathological Clues to How the SARS Virus Kills

**DOI:** 10.1371/journal.pmed.0030059

**Published:** 2006-01-03

Severe acute respiratory syndrome (SARS) first emerged in Guangdong Province, China, in November 2002. At the end of February 2003, an infected doctor from the province inadvertently took the illness to Hong Kong. From there, a woman staying in the same hotel contracted the disease and took it back with her when she returned to Toronto, Canada. SARS, with its ability to spread by close person-to-person contact and with its 10% death rate, was on the move and was threatening to cause a worldwide epidemic. The World Health Organization responded rapidly to this threat by issuing a global alert, and warning against unnecessary travel to affected areas. This and rigorous local containment efforts meant that only 8,098 people became ill, and only 774 people died in this first SARS epidemic.

The last case of the epidemic was reported in Taiwan in June 2003, and since then there have only been a few isolated cases. But the coronavirus responsible for SARS (SARS-CoV) could cause another epidemic at any time. Consequently, scientists continue to study SARS intensively, including researchers from Hong Kong and Toronto, who have joined forces to examine the organs of people who died from SARS in these two cities and now report their results. As John Nicholls, the leader of the international team, explains, understanding how the SARS virus kills people should help in the treatment of SARS if it re-emerges.

Nicholls and colleagues collected post-mortem material from 32 fatal cases of SARS (the largest such collection to date), and asked three questions about the pathogenesis of SARS. First, was SARS-CoV present in the lungs of these patients throughout their illness? Second, which cells in the lungs contained the virus? Third, did any other tissues contain SARS-CoV? The researchers used three molecular techniques to look for SARS-CoV in their specimens: immunohistochemistry, which detects virus-specific proteins; in situ hybridization, which detects the viral genome; and reverse-transcriptase polymerase chain reaction (RT-PCR), which measures viral load.

The researchers found that SARS-CoV was present only in the lungs of patients who died within two weeks of becoming ill (four out of seven patients). In 25 patients who died more than two weeks after the onset of symptoms (generally high temperature and lower respiratory tract symptoms, followed by pneumonia), there was no SARS-CoV in post-mortem lung tissue, although in one case the virus had been present in an open lung biopsy taken five days after disease onset. The researchers found no virus in tissues other than the lung in any of the patients, but in the four patients whose lungs contained SARS-CoV, the virus was found in pneumocytes—cells that line the alveoli, the terminal air spaces where gas exchange occurs—and sometimes in alveolar macrophages, a type of immune cell. No SARS-CoV was found in the cells lining the tubes leading to the alveoli, which explains why patients with SARS only have lower respiratory tract symptoms. [Fig pmed-0030059-g001]

**Figure pmed-0030059-g001:**
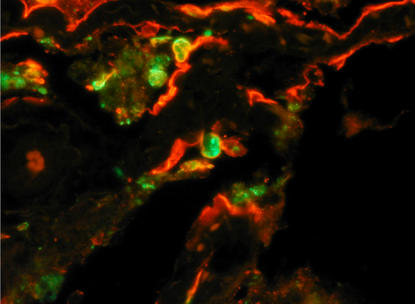
SARS coronavirus (green) in lung cells

These results indicate that the human immune system can stop SARS-CoV replicating within two weeks of infection. By that time, however, the damage to the lungs in some patients appears to be so great that they die even without continued viral replication. This time course of events indicates that antiviral drugs are likely to be useful only during the early stages of SARS. In addition, the absence of virus outside the lungs suggests that death is the result of SARS-CoV replicating in the lungs alone. Whether SARS-CoV fatally damages lung tissue directly or whether macrophages recruited to the lungs in response to infection with SARS-CoV cause fatal immunopathological changes remains an open question.

